# Supplementary material for: The BAF chromatin remodeler synergizes with RNA polymerase II and transcription factors to evict nucleosomes
Source: Nat Genet. 2023 Dec 4;56(1):100–11. doi: 10.1038/s41588-023-01603-8 (PMC10786724; doi:10.1038/s41588-023-01603-8)
Supplement: Supplementary file 1 — Reporting Summary [file 41588_2023_1603_MOESM1_ESM.pdf]

nature portfolio

Corresponding author(s): Steven Henikoff, Sandipan Brahma

Last updated by author(s): Oct 14, 2023

Reporting Summary

Nature Portfolio wishes to improve the reproducibility of the work that we publish. This form provides structure for consistency and transparency in reporting. For further information on Nature Portfolio policies, see our Editorial Policies and the Editorial Policy Checklist.

Statistics

For all statistical analyses, confirm that the following items are present in the figure legend, table legend, main text, or Methods section.

n/a	Confirmed	
☐	☒	The exact sample size (*n*) for each experimental group/condition, given as a discrete number and unit of measurement
☐	☒	A statement on whether measurements were taken from distinct samples or whether the same sample was measured repeatedly
☒	☐	The statistical test(s) used AND whether they are one- or two-sided<br>*Only common tests should be described solely by name; describe more complex techniques in the Methods section.*
☒	☐	A description of all covariates tested
☒	☐	A description of any assumptions or corrections, such as tests of normality and adjustment for multiple comparisons
☐	☒	A full description of the statistical parameters including central tendency (e.g. means) or other basic estimates (e.g. regression coefficient) AND variation (e.g. standard deviation) or associated estimates of uncertainty (e.g. confidence intervals)
☒	☐	For null hypothesis testing, the test statistic (e.g. *F*, *t*, *r*) with confidence intervals, effect sizes, degrees of freedom and *P* value noted<br>*Give P values as exact values whenever suitable.*
☒	☐	For Bayesian analysis, information on the choice of priors and Markov chain Monte Carlo settings
☒	☐	For hierarchical and complex designs, identification of the appropriate level for tests and full reporting of outcomes
☒	☐	Estimates of effect sizes (e.g. Cohen's *d*, Pearson's *r*), indicating how they were calculated

Our web collection on statistics for biologists contains articles on many of the points above.

Software and code

Policy information about availability of computer code

Data collection	The size distributions and molar concentration of libraries were determined using an Agilent 4200 TapeStation. Up to 96 barcoded libraries were pooled at approximately equimolar concentration for sequencing. Paired-end 25×25 bp sequencing on the Illumina HiSeq 2500 platform or PE 50x50 bp sequencing on the Illumina NextSeq 2000 was performed by the Fred Hutchinson Cancer Center Genomics Shared Resources. This yielded 3-6 million reads per antibody/sample. Paired-end reads were aligned using Bowtie2 version 2.3.4.3 to UCSC mm10 with options: --very-sensitive-local --soft-clipped-unmappedtlen --dovetail --no-mixed --no-discordant -q --phred33 -I 10 -X 1000 (for CUT&Tag and CUTAC) or --end-to-end --very-sensitive --no-mixed --no-discordant -q --phred33 -I 10 -X 700 (for CUT&RUN.ChIP). EVOS FL Auto 2 Cell Imaging System (Invitrogen) was used for immunofluroscence imaging. Western blotting images were acquired using Li-Cor Odyssey Dlx Imaging System (LI-COR Biosystems)
Data analysis	Bowtie 2; bedtools v2.30.0; Integrated Genome Browser v 8.5.4; SEACR v.1.3; deeptoolsv 3.5.1; GraphPad Prism 9; ImageJ version 1.53t 24; No custom codes were used.

For manuscripts utilizing custom algorithms or software that are central to the research but not yet described in published literature, software must be made available to editors and reviewers. We strongly encourage code deposition in a community repository (e.g. GitHub). See the Nature Portfolio guidelines for submitting code & software for further information.

Data

Policy information about availability of data

All manuscripts must include a data availability statement. This statement should provide the following information, where applicable:

- Accession codes, unique identifiers, or web links for publicly available datasets
- A description of any restrictions on data availability
- For clinical datasets or third party data, please ensure that the statement adheres to our policy

All primary sequencing data have been deposited as paired-end fastq files and all mapped data have been deposited as bigWig files in the Gene Expression Omnibus under the accession number GSE 224292.
Public datasets used: ATAC-seq: GSM2267967; START RNA-seq: GSM1551910; MNase seq: GSE117767; mESC Enhancer annotation: Whyte et al., 2013

Human research participants

Policy information about studies involving human research participants and Sex and Gender in Research.

Reporting on sex and gender	n/a
Population characteristics	n/a
Recruitment	n/a
Ethics oversight	n/a

Note that full information on the approval of the study protocol must also be provided in the manuscript.

Field-specific reporting

Please select the one below that is the best fit for your research. If you are not sure, read the appropriate sections before making your selection.

☒ Life sciences ☐ Behavioural & social sciences ☐ Ecological, evolutionary & environmental sciences

For a reference copy of the document with all sections, see nature.com/documents/nr-reporting-summary-flat.pdf

Life sciences study design

All studies must disclose on these points even when the disclosure is negative.

Sample size	In this study we collected chromatin profiling data for comparative analysis from 2 to 3 replicate populations of cells for each condition, with cell numbers from 50,000 - 1,000,000, depending on specific method requirements. Each sample was sequenced to a depth of 3-6 million reads, sufficient for bulk characterization of chromatin in each sample. These sequencing depths and replicate numbers are standard in the field.
Data exclusions	Sequencing reads mapping to the mitochondrial genome were removed from all datasets. This was pre-established and is standard practice in the field. The purpose of this study was to perform comparative analysis of chromatin profiles from the nuclear genome and this can be confounded by variable read numbers from the mitochondrial genome.
Replication	At least 2 biological replicates were profiled. All attempts at replication were successful.
Randomization	n/a. These studies compare the same kinds of cells with and without experimental treatment under laboratory conditions, and data and analysis for this study are objective and not prone to influence by the researchers bias.
Blinding	n/a. The data and analysis for this study is objective and not prone to influence by researchers bias.

Reporting for specific materials, systems and methods

We require information from authors about some types of materials, experimental systems and methods used in many studies. Here, indicate whether each material, system or method listed is relevant to your study. If you are not sure if a list item applies to your research, read the appropriate section before selecting a response.

Materials & experimental systems

n/a	Involved in the study
☐	☒ Antibodies
☐	☒ Eukaryotic cell lines
☒	☐ Palaeontology and archaeology
☒	☐ Animals and other organisms
☒	☐ Clinical data
☒	☐ Dual use research of concern

Methods

n/a	Involved in the study
☐	☒ ChIP-seq
☒	☐ Flow cytometry
☒	☐ MRI-based neuroimaging

Antibodies

Antibodies used

RNAPII-S5P: rabbit monoclonal (D9N5I, Cell Signaling Technology cat. no. 13523), 1:50; RNAPII-S2P: rabbit monoclonal (E1Z3G, Cell Signaling Technology cat. no. 13499), 1:100; RPB3: rabbit polyclonal (Bethyl Laboratories cat. no. A303-771A, lot no. A303-771A2), 1:100; BRG1: rabbit monoclonal (EPNCIR111A, abcam cat. no. ab110641), 1:100, for CUT&Tag and CUT&RUN.ChIP, and rabbit polyclonal (Invitrogen cat. no. 720129, lot. no. 2068859), 1:250, for immunofluorescence; H3K4me1: rabbit polyclonal (Abcam cat. no. ab8895, lot no. GR3283237), 1:100; H3K4me3: rabbit polyclonal (Active Motif cat. no. 39915, lot. no. 24118008), 1:100, for CUT&Tag, and rabbit monoclonal (EpiCypher cat. no. 13-0028), 1:100, for CUT&RUN.ChIP; H3K27me3: rabbit monoclonal (C36B11, Cell Signaling Technology cat. no. 9733), 1:100; H3K9me3: rabbit monoclonal (EPR16601, Abcam cat. no. ab176916), 1:100; guinea pig anti-rabbit secondary: Antibodies Online cat. no. ABIN101961, 1:100; isotype control (IgG) for CUT&RUN.ChIP: rabbit monoclonal (EPR25A, Abcam cat. no. ab172730), 1:100; NANOG:¬¬¬ rabbit polyclonal (Bethyl Laboratories cat. no. A300-397A, lot no. 3), 1:100; KLF4: goat polyclonal (R&D Systems cat. no. AF3158, lot. no. WRR0719011), 1:100 for CUT&RUN, 1:50 for immunofluorescence; OCT4: rabbit monoclonal (EPR17929, Abcam cat. no. ab181557), 1:100; SOX2: rabbit monoclonal (EPR3131, abcam cat. no. ab92494), 1:100; CTCF: rabbit monoclonal (EPR7314(B), Abcam cat. no. ab128873), 1:100; rabbit anti-goat secondary: Abcam cat. no. ab6697, 1:100; goat anti-rabbit-Cy5 secondary: Jackson ImmunoResearch Cat. no. 111-175-144, 1:500; donkey anti-goat-rhodamine red secondary: Jackson ImmunoResearch Cat. no. 705-295-147, 1:250; mouse anti-H3 for Western blot: (mAbcam 24834, Abcam cat. no. ab24834), 1:500; IRDye 800CW goat anti-rabbit: LI-COR INC. Cat no. 926-32211, 1:10,000; IRDye 800CW donkey anti-goat: LI-COR INC. Cat no. 926-32214, 1:10,000; IRDye 680RD goat anti-mouse: LI-COR INC. Cat no. 926-68070, 1:10,000.

Validation

All antibodies were sourced commercially. RNAPII-S5P (CST 13523) and RNAPII-S2P (CST 13499) antibodies were validated by Cell Signaling Technologies using SimpleChIP® Enzymatic Chromatin IP Kits, and do not cross react.
BRG1 antibody (ab110641) was knock-out validated by abcam.
CTCF antibody (ab128873) was ChIP-Seq validated with ChIP-Kit Transcription Factors ChIP-Seq (ab270813) by abcam.

Eukaryotic cell lines

Policy information about cell lines and Sex and Gender in Research

Cell line source(s)

AB2.2 (Male; strain: 129S5/SvEvBrd) mouse embryonic stem cells were used in all experiments. Frozen stock of cells were obtained from ATCC (ATCC SCRC-1023).

Authentication

Cell cultures were tested for Mycoplasma and karyotyped to detect any chromosomal abnormalities after thawing and at periodic intervals. Cells were not cultured for more than seven passages for any experiment.

Mycoplasma contamination

All cell lines were confirmed as Mycoplasma negative on a tri-monthly basis.

Commonly misidentified lines
(See ICLAC register)

No commonly misidentified lines were used in this study.

ChIP-seq

Data deposition

☒ Confirm that both raw and final processed data have been deposited in a public database such as GEO.

☒ Confirm that you have deposited or provided access to graph files (e.g. BED files) for the called peaks.

Data access links
May remain private before publication.

https://urldefense.com/v3/__https://www.ncbi.nlm.nih.gov/geo/query/acc.cgi?acc=GSE224292__;!!GuAltXPztq0!mkMYN8s5c8cDPzIRwHc7Ok6WhX3or4EV-xQ1CZoZO73zAker5r1qbMQbfa7gjsZlHviPdoygyAUjG1E5$
(reviewer token: qvkzowmodnithgf)

Files in database submission

RNAPII-S5P_CUTAC_SL_0(SB_Mm_050222_SL_0_CUTAC)
RNAPII-S5P_CUTAC_SL_FLV_8h(SB_Mm_050222_SL_FL8_CUTAC)
RNAPII-S5P_CUTAC_SL(SB_Mm_SL_0506_0_S5_CUTAC)
RNAPII-S5P_CUTAC_2i(SB_Mm_2i_0506_0_S5_CUTAC)
RNAPII-S5P_CUTnTag_SL_DMSO(SB_Mm_ABSL_032421_0_S5P)
RNAPII-S5P_CUTnTag_SL_TRP_30m(SB_Mm_ABSL_032421_TR30_S5P)
RNAPII-S5P_CUTnTag_SL_TRP_1h(SB_Mm_ABSL_032421_TR1_S5P)
RNAPII-S5P_CUTnTag_SL_TRP_2h(SB_Mm_ABSL_032421_TR2_S5P)

RNAPII-S5P_CUTnTag_SL_DMSO_2(SB_MmDmEc_0715_0_S5P)
RNAPII-S5P_CUTnTag_SL_FLV_30m(SB_MmDmEc_0715_F30_S5P)
RNAPII-S5P_CUTnTag_SL_FLV_1h(SB_MmDmEc_0715_F1_S5P)
RNAPII-S5P_CUTnTag_SL_FLV_4h(SB_MmDmEc_0715_F2_S5P)
RNAPII-S5P_CUTnTag_SL_ACT_30m(SB_MmDmEc_0715_A30_S5P)
RNAPII-S5P_CUTnTag_SL_ACT_1h(SB_MmDmEc_0715_A1_S5P)
RNAPII-S5P_CUTnTag_SL_ACT_4h(SB_MmDmEc_0715_A4_S5P)
BRG1_CUTnTag_SL_DMSO(SB_Mm_ABSL_032421_0_BRG)
BRG1_CUTnTag_SL_TRP_30m(SB_Mm_ABSL_032421_TR30_BRG)
BRG1_CUTnTag_SL_TRP_1h(SB_Mm_ABSL_032421_TR1_BRG)
BRG1_CUTnTag_SL_TRP_2h(SB_Mm_ABSL_032421_TR2_BRG)
BRG1_CUTnTag_SL_DMSO_2(SB_MmDmEc_0715_0_BRG1)
BRG1_CUTnTag_SL_FLV_30m(SB_MmDmEc_0715_F30_BRG1)
BRG1_CUTnTag_SL_FLV_1h(SB_MmDmEc_0715_F1_BRG1)
BRG1_CUTnTag_SL_FLV_4h(SB_MmDmEc_0715_F4_BRG1)
BRG1_CUTnTag_SL_ACT_30m(SB_MmDmEc_0715_A30_BRG1)
BRG1_CUTnTag_SL_ACT_1h(SB_MmDmEc_0715_A1_BRG1)
BRG1_CUTnTag_SL_ACT_4h(SB_MmDmEc_0715_A4_BRG1)
RNAPII-S5P_CUTnTag_SL(SB_Mm_AB2SL_0319_S5P)
RNAPII-S5P_CUTnTag_2i(SB_Mm_AB2S2i_0319_S5P)
BRG1_CUTnTag_SL(SB_Mm_AB2SL_0319_BRG1)
BRG1_CUTnTag_2i(SB_Mm_AB2S2i_0319_BRG1)
RNAPII-S2P_CUTnTag_SL(SB_MmDmEc_0715_0_S2P)
RNAPII-RPB3_CUTnTag_SL(TL_Mm_AB22_DMSO_Rpb3_TL2_122021)
H3K4me1_CUTnTag_SL(SB_Mm_AB2SL_0319_K4m1)
H3K4me3_CUTnTag_SL(SB_Mm_AB2SL_0319_K4m3)
H3K27me3_CUTnTag_SL(SB_Mm_AB2SL_0319_K27m3)
H3K27me3_CUTnTag_SL_DMSO(SB_Mm_0625_0_K27m)
H3K27me3_CUTnTag_SL_FLV_8h(SB_Mm_0625_FL8_K27m)
H3K9me3_CUTnTag_SL(SB_Mm_G4X40_0514_K9m3)
IgG_CUTnTag_SL(SB_Mm_AB2SL_0319_IgG)
BRG1_CUTnRUN_SL_DMSO(SB_MmSc_0620_0_Brg_in)
BRG1_H3K4me1_CUTnRUN.ChIP_SL_DMSO(SB_MmSc_0620_0_Brg_K4m1)
BRG1_H3K4me3_CUTnRUN.ChIP_SL_DMSO(SB_MmSc_0620_0_Brg_K4m3)
BRG1_H3K27me3_CUTnRUN.ChIP_SL_DMSO(SB_MmSc_0620_0_Brg_K27m3)
BRG1_IgG_CUTnRUN.ChIP_SL_DMSO(SB_MmSc_0620_0_Brg_IgG)
BRG1_CUTnRUN_SL_FLV(SB_MmSc_0620_FL8_Brg_in)
BRG1_H3K4me1_CUTnRUN.ChIP_SL_FLV(SB_MmSc_0620_FL8_Brg_K4m1)
BRG1_H3K4me3_CUTnRUN.ChIP_SL_FLV(SB_MmSc_0620_FL8_Brg_K4m3)
BRG1_H3K27me3_CUTnRUN.ChIP_SL_FLV(SB_MmSc_0620_FL8_Brg_K27m3)
BRG1_IgG_CUTnRUN.ChIP_SL_FLV(SB_MmSc_0620_FL8_Brg_IgG)
BRG1_CUTnRUN_SL(SB_MmScEc_SL_1217_BRG1_in)
BRG1_H3K4me1_CUTnRUN.ChIP_SL(SB_MmScEc_SL_1217_BRG1_K4m1)
BRG1_H3K4me3_CUTnRUN.ChIP_SL(SB_MmScEc_SL_1217_BRG1_K4m3)
BRG1_H3K27me3_CUTnRUN.ChIP_SL(SB_MmScEc_SL_1217_BRG1_K27m3)
BRG1_IgG_CUTnRUN.ChIP_SL(SB_MmScEc_SL_1217_BRG1_IgG)
BRG1_CUTnRUN_2i(SB_MmScEc_2i_1217_BRG1_in)
BRG1_H3K4me1_CUTnRUN.ChIP_2i(SB_MmScEc_2i_1217_BRG1_K4m1)
BRG1_H3K4me3_CUTnRUN.ChIP_2i(SB_MmScEc_2i_1217_BRG1_K4m3)
BRG1_H3K27me3_CUTnRUN.ChIP_2i(SB_MmScEc_2i_1217_BRG1_K27m3)
BRG1_IgG_CUTnRUN.ChIP_2i(SB_MmScEc_2i_1217_BRG1_IgG)
NANOG_CUTnRUN_SL(SB_MmScEc_SL_1217_NAN2)
SOX2_CUTnRUN_SL(SB_MmScEc_SL_1217_Sox2)
OCT4_CUTnRUN_SL(SB_MmScEc_SL_1217_Oct4)
KLF4_CUTnRUN_SL(SB_MmScEc_SL_1217_Klf4)
IgG_CUTnRUN_SL(SB_MmScEc_SL_1217_IgG)
NANOG_CUTnRUN_2i(SB_MmScEc_2i_1217_NAN2)
SOX2_CUTnRUN_2i(SB_MmScEc_2i_1217_Sox2)
OCT4_CUTnRUN_2i(SB_MmScEc_2i_1217_Oct4)
KLF4_CUTnRUN_2i(SB_MmScEc_2i_1217_Klf4)
IgG_CUTnRUN_2i(SB_MmScEc_2i_1217_IgG)

Genome browser session
(e.g. UCSC)

Integrated Genome Browser (IGB) sessions:
Fig 3: https://drive.google.com/file/d/1uu7zFtg4d2JqvuKoCBNtkaZA1-9dEyWG/view?usp=sharing
Fig 4: https://drive.google.com/file/d/1hj-vvZl7TjUfLijV71gBl-gvGUcVXvTl/view?usp=sharing
Extended Data Fig 1: https://drive.google.com/file/d/1cVAHxUt5iPaRDkBi1P273aWUHcCXorna/view?usp=sharing

Methodology

Replicates

At least 2 biological replicates for each dataset were performed. Timepoints during drug treatment provide additional validation. All experiments were reproducible and replicates were consistent.

Sequencing depth

All Experiments were paired-end sequenced for 3-6 million reads

Antibodies	RNAPII-S5P: rabbit monoclonal (D9N5I, Cell Signaling Technology cat. no. 13523), 1:50; RNAPII-S2P: rabbit monoclonal (E1Z3G, Cell Signaling Technology cat. no. 13499), 1:100; RPB3: rabbit polyclonal (Bethyl Laboratories cat. no. A303-771A, lot no. A303-771A2), 1:100; BRG1: rabbit monoclonal (EPNCIR111A, abcam cat. no. ab110641), 1:100, for CUT&Tag and CUT&RUN.ChIP, and rabbit polyclonal (Invitrogen cat. no. 720129, lot. no. 2068859), 1:250, for immunofluorescence; H3K4me1: rabbit polyclonal (Abcam cat. no. ab8895, lot no. GR3283237), 1:100; H3K4me3: rabbit polyclonal (Active Motif cat. no. 39915, lot. no. 24118008), 1:100, for CUT&Tag, and rabbit monoclonal (EpiCypher cat. no. 13-0028), 1:100, for CUT&RUN.ChIP; H3K27me3: rabbit monoclonal (C36B11, Cell Signaling Technology cat. no. 9733), 1:100; H3K9me3: rabbit monoclonal (EPR16601, Abcam cat. no. ab176916), 1:100; guinea pig anti-rabbit secondary: Antibodies Online cat. no. ABIN101961, 1:100; isotype control (IgG) for CUT&RUN.ChIP: rabbit monoclonal (EPR25A, Abcam cat. no. ab172730), 1:100; NANOG:¬¬¬ rabbit polyclonal (Bethyl Laboratories cat. no. A300-397A, lot no. 3), 1:100; KLF4: goat polyclonal (R&D Systems cat. no. AF3158, lot. no. WRR0719011), 1:100 for CUT&RUN, 1:50 for immunofluorescence; OCT4: rabbit monoclonal (EPR17929, Abcam cat. no. ab181557), 1:100; SOX2: rabbit monoclonal (EPR3131, abcam cat. no. ab92494), 1:100; CTCF: rabbit monoclonal (EPR7314(B), Abcam cat. no. ab128873), 1:100; rabbit anti-goat secondary: Abcam cat. no. ab6697, 1:100; goat anti-rabbit-Cy5 secondary: Jackson ImmunoResearch Cat. no. 111-175-144, 1:500; donkey anti-goat-rhodamine red secondary: Jackson ImmunoResearch Cat. no. 705-295-147, 1:250; mouse anti-H3 for Western blot: (mAbcam 24834, Abcam cat. no. ab24834), 1:500; IRDye 800CW goat anti-rabbit: LI-COR INC. Cat no. 926-32211, 1:10,000; IRDye 800CW donkey anti-goat: LI-COR INC. Cat no. 926-32214, 1:10,000; IRDye 680RD goat anti-mouse: LI-COR INC. Cat no. 926-68070, 1:10,000.
Peak calling parameters	SEACR v 1.3 was used for peak calling. Parameters are described in the Methods section.
Data quality	SEACR peaks were called using default FDR and "relaxed" parameter
Software	Bowtie 2 was used to clip adapters post sequencing and map paired-end Mus musculus reads to UCSC mm10, and spike-in E. coli reads to Ensembl masked R64-1-1. Continuous-valued data tracks (bedGraph and bigWig) were generated using genomecov in bedtools v2.30.0 Genomic tracks were displayed using Integrated Genome Browser v 8.5.4. Peaks were called by SEACR (v.1.3). Profile plots, heatmaps, and correlation matrices were generated using deepTools v3.5.1. Violin plots were generated with GraphPad Prism 9, scores were computed using deepTools v3.5.1. Western blot images were developed and bands quantified using ImageJ version 1.53t 24.
