# Supplementary figures and images for: The BAF chromatin remodeler synergizes with RNA polymerase II and transcription factors to evict nucleosomes
Source: Nat Genet. 2023 Dec 4;56(1):100–11. doi: 10.1038/s41588-023-01603-8 (PMC10786724; doi:10.1038/s41588-023-01603-8)

# Unprocessed blots for Extended Data Fig. 5

SOX2

BRG1

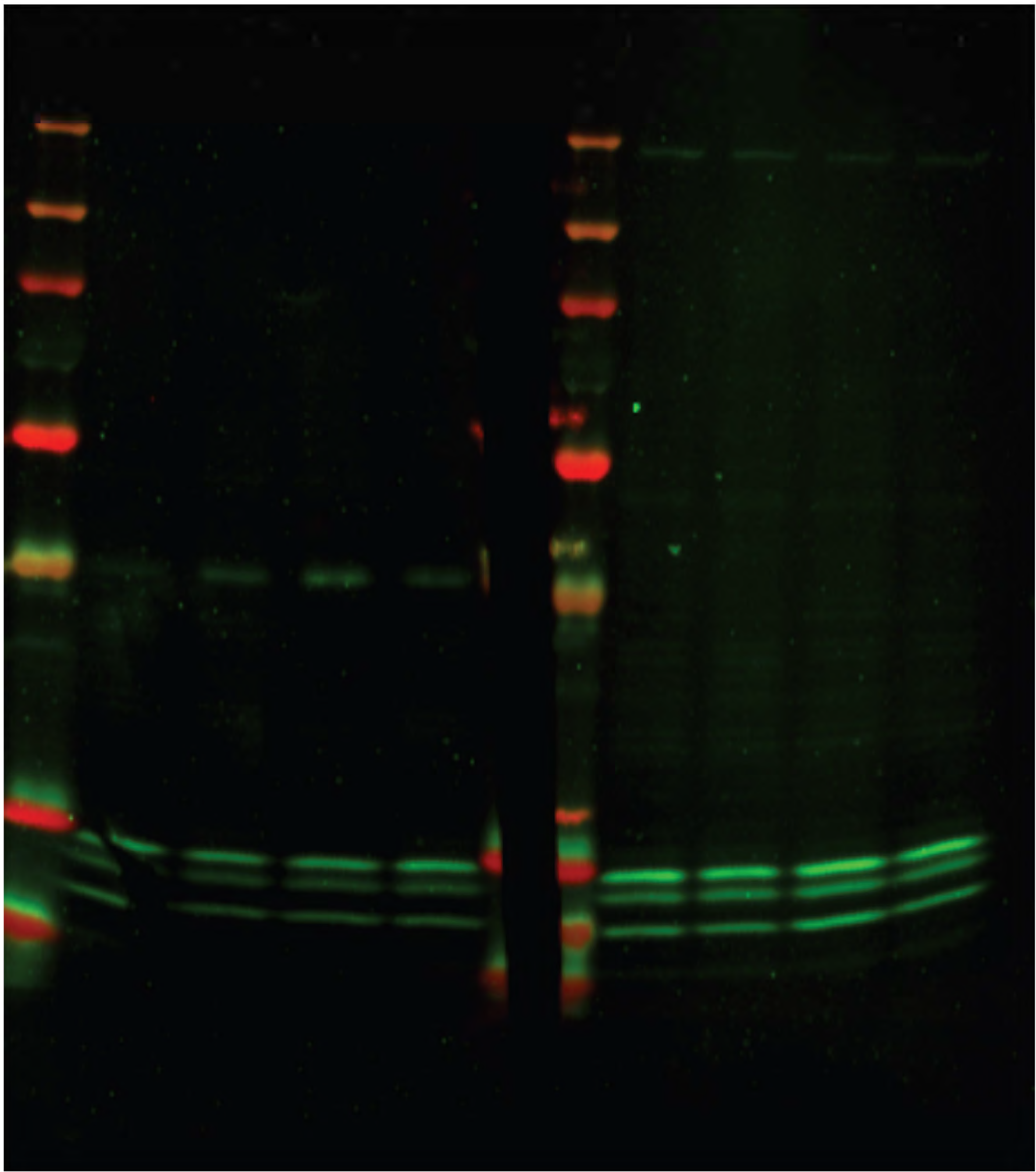

NANOG

OCT4

KLF4

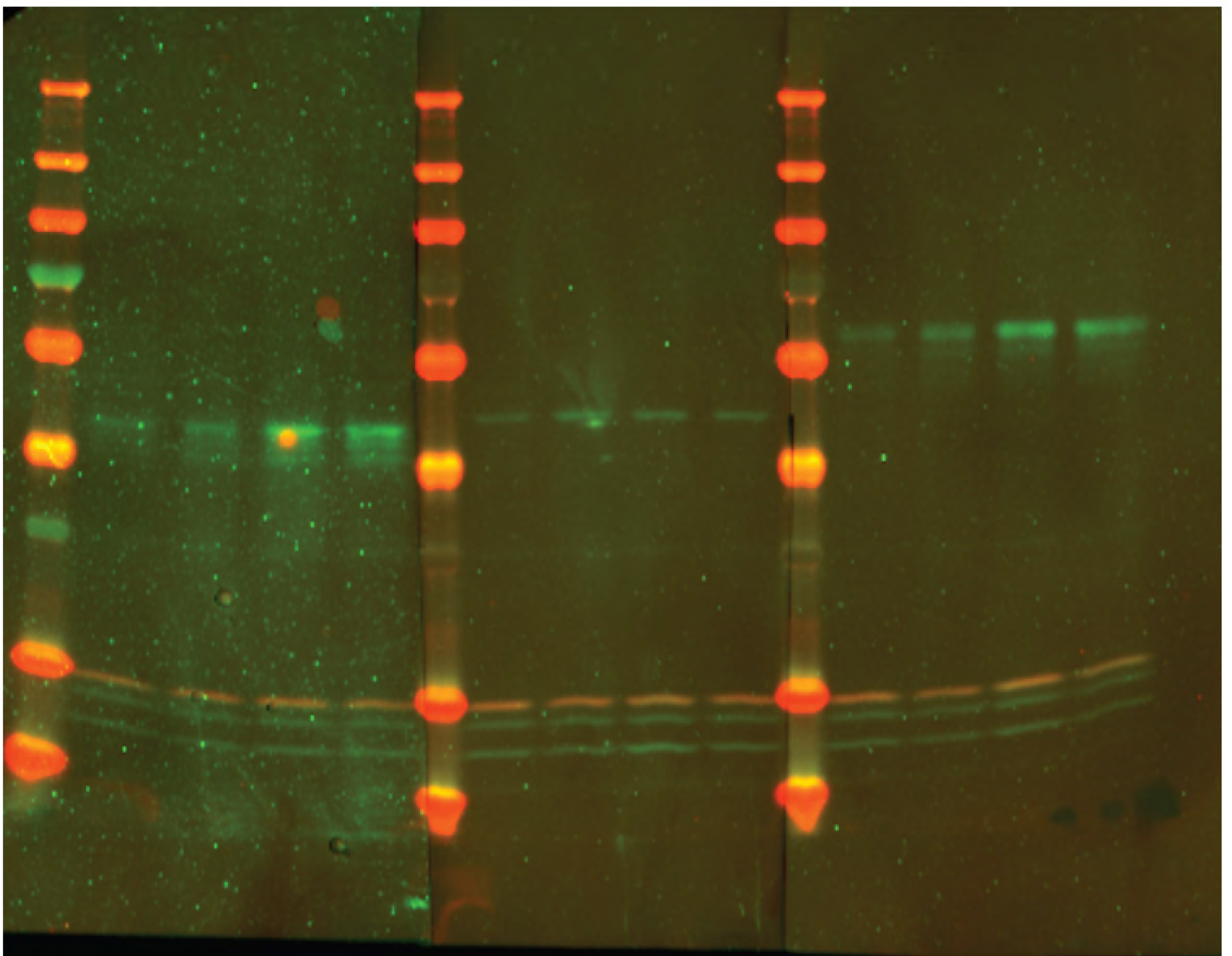

Supplement: Supplementary file 4 — Unprocessed western blot images. [file 41588_2023_1603_MOESM4_ESM.pdf]
